# The Effect of Opium Dependency of Parent (s) on Offspring’s Spatial Learning & Memory in Adult Male Rats

**Published:** 2013-05

**Authors:** Arezoo Saberi Moghadam, Gholamreza Sepehri, Vahid Sheibani, Tahereh Haghpanah, Kouros Divsalar, Mousa-Al-Reza Hajzadeh, Mohammadreza Afarineshkhaki

**Affiliations:** 1 Kerman Neuroscience Research Center, Kerman University of Medical Sciences Kerman, Iran; 2 Department of Anatomical and Reproductive Biology, Faculty of Medical sciences, Shahid Beheshti University of Medical Sciences, Tehran, Iran.; 3 Mashhad Cognitive Neuroscience Research Center and Department of Physiology, Faculty of Medical sciences, Mashhad University of Medical Sciences, Mashhad, Iran; 4 Department of Physiology and Medical Student Research Committee, Faculty of Medical sciences, Shahid Beheshti University of Medical Sciences. Tehran, Iran

**Keywords:** Learning, Memory, Opium, Parent (s) dependency, Rat

## Abstract

***Objective(s):*** As far as we know, there has been no report regarding the effects of opium addiction or dependency of both parents on the learning and memory process in offspring. The aim of this study was to examine the learning and memory changes of adult male offspring whose mothers, fathers and/or both parents had dependency to opium before and during pregnancy.

***Materials and Methods*** *:* All experiments were carried out on Wistar rats. Opium dependency was induced by daily injections of opium (10 mg/kg/SC, bid/10 d) before mating. The presence of a vaginal plug was designated as gestation day. Treatment with opium continued through breeding and gestation until parturition. Spatial memory was tested in male offspring of control, saline and prenatal opium treated groups by a training trial and the probe test in the Morris water maze. Swimming escape latency in the maze and the ability to find the platform in the training trial were recorded. The time spent in the trigger zone and number of times the rats crossed the platform during the probe phase and swimming speed were measured.

***Results:*** The data revealed increased escape latency and a greater distance traveled to find the hidden platform in the offspring’s whose mother, father and /or both parents were exposed to opium. Crossings to target quadrant at probe trials was significantly reduced in all of the prenatal opium exposed offsprings. The swimming speed showed a significant increase in father and parent’s opium exposed offspring.

***Conclusion:*** Prenatal opium exposure of either parent may cause deficits in spatial learning, but the precise mechanism(s) remain largely unknown.

## Introduction

Opioid receptors are found in different regions of the central nervous system and in animal studies, considerable evidence has shown that the developing opioid system can undergo long-lasting changes by prenatal morphine exposure (1).

Prenatal morphine exposure can produce long-term changes in the opiate system (2, 3), which modulates neural processes that are essential to memory consolidation (4). Prenatal morphine exposure may alter the capacity for learning and memory in adults (5, 6). Sarkaki *et al* (2008) reported that both parental and paternal addiction to morphine may cause memory deficiency through reduction of LTP in the hippocampus (5). Heroin abuse during pregnancy has long been known to increase the risk of a variety of neurobehavioral defects in the offspring (7, 8). 

Unlike the pure opioids such as morphine and heroin, opium is a complex and variable mixture of substances reflecting differences in both the starting material and the traditional practices of the regions in which it is produced (9). The major constituents of opium are morphine (about 10% by weight), noscapine (about 6%), papaverine (about 1%), codeine (about 0.5%) and thebaine (about 0.2%)(10). 

Opium abuse is a global problem and is used by many young people in many eastern countries, including Iran. Opium abuse furthers socio-economic and political instability, it undermines sustainable development, and it hampers efforts to reduce poverty and crime (11). Although heroin is one of the most common opioid drugs used by pregnant women in many countries, however, opium abuse is also very popular among young Iranian men and women, the country in which the highest amount of opium is abused. Drug abuse during pregnancy may result in many serious damages to the offspring, including cognitive functions, social behaviors, and addictive susceptibility (12, 13).

Most of the previous studies were focused on mother’s exposure to pure opioids such as morphine, heroin and codeine during pregnancy and their ability to alter many biological functions in offspring, including learning and memory process (5, 6, 14, 15), however, there has been no report regarding the effect of opium addiction or dependency of both parents on the learning and memory process in offspring, so the aim of this study was to examine the extent of learning and memory deficits in adult male offspring whose mothers , fathers and/or both parents abused opium during pregnancy.

## Materials and Methods

All experiments were carried out on Wistar rats, weighing 275-300 g, obtained from Kerman Neuroscience Animal House and were housed four per cage under a 12 hr light/dark cycle in a room under controlled temperature (23±2°C). Food and water were available *ad libitum*. Sperm positive dams were randomly assigned to test (opium), saline or control groups, each comprised of 7 animals. All of the procedures were in accordance with guidelines of the Neuroscience Research Center of Kerman University of Medical Sciences and the Neuroscience Ethic Committee (EC/KNRC/88-24) for care and use of laboratory animals.

***Induction of dependency***

Opium dependency was induced by daily injections of aqueous opium solution (10 mg/kg/SC, two times per day) for 10 days before mating. Opium was obtained from the campaign office against narcotics, which is a military security organization and it was the purest form of opium which was discovered and kept by this organization. Naloxone (2 mg/kg/IP) was injected randomly to some rats to prove the dependency by the expression of opioid withdrawal signs (16).

During breeding, two males were housed with five females. The presence of a vaginal plug was designated as gestation day (E0). Sperm positive females were removed from the breeding cages and housed individually in nesting boxes with *ad libitum* food and water.

Treatment with opium continued through breeding and gestation until parturition. Thereafter the opium dose was gradually decreased to prevent the signs of opiate withdrawal. The presence of opiate withdrawal can confound data interpretation. Thus, withdrawal signs such as weight loss, diarrhea, and irritability were monitored daily. Male pups were reared by their biological mothers, weaned at postnatal day 25, and housed with like-treated male subjects. The exposed offspring remained undisturbed until testing commenced for two months and only one adult male rat was selected from each mother for behavioral testing (16).

 Male rat offspring were randomly divided into 5 groups:

1. Control group: offspring of untreated males and females
2. Saline group: offspring of female rats that had received saline
3. Test group 1: offspring of opium exposed females
4. Test group 2: offspring of opium exposed males
5. Test group 3: offspring of both parent opium exposed

***Apparatus***

Spatial memory was tested by the Morris water maze (MWM) apparatus. The Morris water maze (MWM) was a black circular pool with a diameter of 150 cm and a height of 60 cm, filled with 21 ± 1 °C of water to a depth of 25 cm. The maze was divided geographically into four equal quadrants and release points were designed at each quadrant as N, E, S, and W. A hidden circular platform (10 cm in diameter), made of Plexiglass, was located in the center of the southwest quadrant, submerged 1.5 cm beneath the surface of the water. Fixed, extra maze visual cues were present at various locations around the maze (i.e., computer, MWM hardwares, posters). A camera was mounted above the center of the maze and animal motion was recorded and sent to the computer. A tracking system by a commercial software (Noldus, Netherlands; version:6 XT) was used to measure the escape latency, crossings (the number of times the rat swam directly over the location of the platform on training and probe trials) and swimming speed (17).

***Behavioral procedure***

1- Habituation: Before orientation training, animals were habituated to the swimming pool environment. They were placed individually on the platform for 20 s and in the pool for 180 s and allowed to swim.

2- Training trial: In the orientation training, mice were trained to escape from drowning by climbing onto the hidden platform; they were allowed to swim for maximum trial duration of 90 s, and were placed on the platform for 30 s (reinforcement) if they failed to find it themselves. Rats were given four trials per day for 4 consecutive days. In each trail, rats were gently placed onto the MWM at the middle of the circular edge in a randomly selected quadrant, with the nose pointing toward the wall. Each training session comprised of four trials, with an inter-trial interval of 60 sec, and was performed routinely between 10:00 AM and 5:00 PM. the intersession interval was 2 hr. The escape latency (i.e. the time required for the rat to find and climb onto the platform) was recorded for each trial. The average of the four trials per training day was recorded.

If a rat failed to find the platform within the testing time or if it stayed on the platform for less than 3 sec (and thus considered to be continuing its search for the target), a score latency of “120 sec” was awarded. After completion of the training, the animals were returned to their home cages until retention testing (probe trial) 24 hr later.

3- Retention testing (Probe trial): Probe test was run 24 hr after the last trial of day 4. In the probe test, the platform was removed from the pool and each rat was placed into the pool from the opposite quadrant. The probe trial consisted of a 60 sec free swim period without a platform and the time swum in the target quadrant was recorded. The crossings to trigger zone and swimming speed of the rats were also recorded.

**Statistical analysis**

Prism software was used for the data analysis. Repeated measures ANOVA was used to analyze the quantitative differences in swimming escape latency in Morris water maze to find the platform. Swimming speed and swimming period duration and number of times the rat crossed the platform in the probe phase (retention testing) was analyzed by one way analysis of variance (ANOVA) followed by Benferroni *post hoc* tests. Data are expressed as mean± SEM of 7 rats per group. *P* values <0.05 was considered statistically significant.

## Results

The results of this study showed that there was no difference in the acquisition parameter of control and saline treated rats, so the result of opium exposed offspring (test groups) were compared to the controls.

1. The effect of prenatal administration of opium on average escape latency of male offspring to find the hidden platform in MWM during the 4 day training.

**Figure 1:**
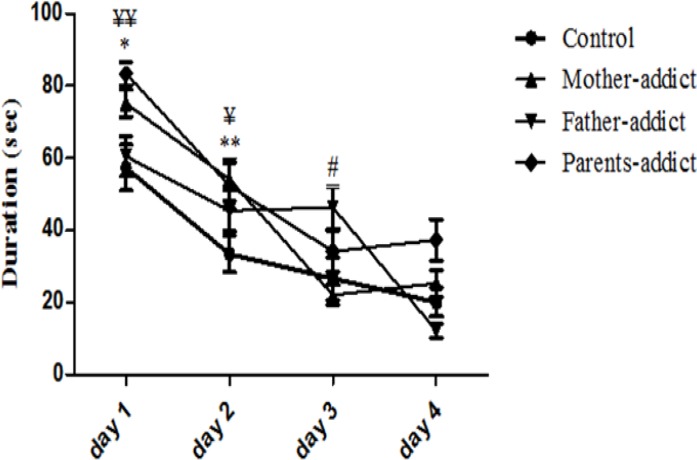
The effect of prenatal administration of opium on average escape latency of the 4 days (16 trials) of training to find hidden platform in the morris water maze in male offspring. Data are expressed as mean±SEM. *P-*values <0.05 was considered statistically significant (n=7)

Data analysis showed a progressive reduction in the escape latency to find the hidden platform in MWM in all control and test groups (*P*>0.0001, F _3,724_= 85.79). The mean escape latency to find hidden platform in MWM was significantly increased in opium exposed offspring (*P*>0.0001, F _3,724_=11.31). Benferroni *post hoc* test showed a significant increase in the escape latency to find the hidden platform in MWM in the mother opium exposed offspring during the first (*P*>0.05) and 2nd day (*P*>0.01), and also father opium exposed offspring during the 3rd day (*P*>0.05), and both parents opium exposed offspring during the first (*P*>0.01) and 2nd day (*P*>0.05) as compared to controls. Data showed a significant correlation between the training trial days and spatial memory (*P*>0.0001, F _15,724_=3.345) (Figure 1).

**Figure 2:**
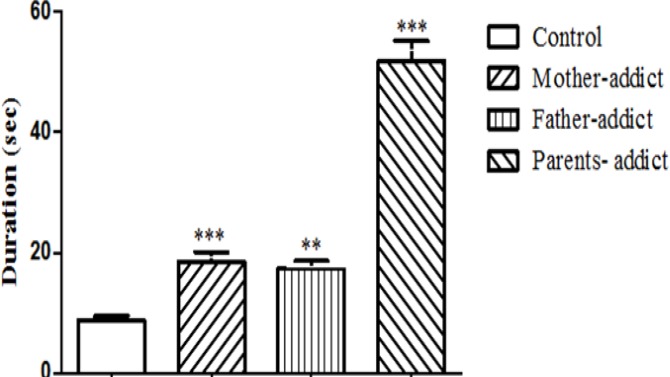
The effect of prenatal administration of opium on total average escape latencies to find the hidden platform during the four days of training in the morris water maze in male offspring. Data are expressed as mean± SEM. *P* values <0.05 was considered statistically significant (n=7)

One way ANOVA followed by *post hoc* (Benferroni test) showed a significant increase in the mean escape latencies to find the hidden platform during the four days of training in the MWM in offspring with mother, father and/or parents (both father & mother) exposed to opium and they spent more time in the MWM to find the hidden platform in comparison with controls (*P*>0.001, *P*>0. 01, *P*>0.001, respectively) (Figure 2). However, the escape latencies of the parents opium exposed offspring showed an increase significant compare to the others (*P*>0.001), while there was no difference significant between the mother and father opium exposed offspring.

1. The effect of prenatal administration of opium on average swimming duration of male offspring to find the hidden platform in the MWM during the probe test (memory retention).

**Figure 3:**
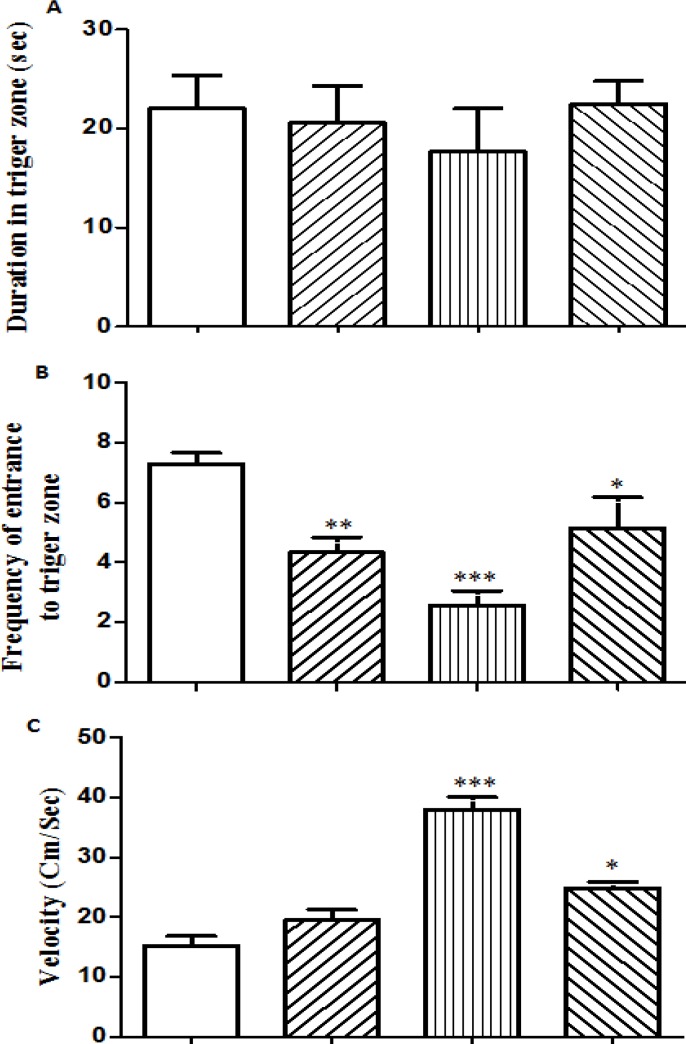
The effect of prenatal administration of opium on the time spent in the trigger zone (A), the number of the rat crossed the platform (B) and swimming speed (C) during probe test (memory retention) to find hidden platform in the morris water maze in adult male exposed offspring.

The time spent in each virtual quadrant of the MWM during the probe test was analyzed by a one way ANOVA (animals × quadrants) for each group. After analyzing the 60 sec period, no significant influence of quadrants appeared for all of the groups and there was no overall effect of prenatal opium exposure on the time spent in the trigger zone to find the hidden platform in male offspring during the probe test (Figure 3A). One way ANOVA followed by *post hoc* (Benferroni test) revealed a significant reduction in crossings to target quadrant for all test group’s offspring (mother, father & both parents opium exposed offspring) versus control during the probe trials (*P*>0.01, *P*>0.001, *P*>0.05, respectively) (Figure 3B). However, there was no significant difference in crossings to target quadrant among prenatal opium exposure groups to find the hidden platform. The swimming speed of the father and both parents opium exposed offspring showed a significant increase during the probe test (*P*>0. 001 and *P*>0.05, respectively) (Figure 3C). However, the swimming speed of the father opium exposed offspring showed a significant increase in comparison with the both parent opium exposed offspring (*P*>0.001), while there was no significant difference between the mother and both parent opium exposed offspring. 

*= *P*>0.05, **= *P*>0.001, Mother opium exposed vs. control

# = *P*>0.05, Father opium exposed vs. control

¥ = *P*>0.05, ¥¥ = *P*>0.01, Parents opium exposed vs. control

**= *P*>0. 01, ***= *P*>0.001, Test groups vs. control group

Data are expressed as mean± SEM. **= *P*>0. 01, Mother opium exposed vs control , ***= *P*>0.001, Father opium exposed vs control, *= *P*>0.05 , Parents opium exposed vs control (n=7)

## Discussion

The findings of this study indicate that prenatal opium dependency in father, mother and/or both parents causes deficits in spatial learning. The spatial learning deficit was characterized by an increased escape latency to find the hidden platform in the prenatal opium-exposed rats. Retention test was conducted on the next day after the final day of training trials. Our results indicate that prenatal opium exposure results in long lasting deficits in spatial reference memory as measured by learning in the MWM. Performance of opium exposed male offspring in the training trials was impaired across the four days of testing relative to controls; however, performance in the probe trials was equal between the groups, but there was a significant reduction in crossings to target quadrant for all test group’s offspring. These deficits in cognitive function persist despite a lengthy period of abstinence from opium exposure. Most of the previous studies focused on mother’s exposure to synthetic opioids, such as morphine, heroin and codeine, during pregnancy and its probable effects on prenatal opioid exposed offspring (5, 6, 14, 15) , however, this is a novel report which shows that prenatal exposure of either mother, father and / or both parents to opium will cause memory deficit in natural opium exposed offspring. Also the probe test showed a significant reduction in crossings to target quadrant in prenatal opium-exposed rats. Sarkaki, *et al* (2008) showed that father morphine exposure causes a clear deficits in maintenance of LTP in offspring (5). Also, it has been shown that father morphine exposure 5 days before mating reduces water maze learning in offspring (18). Father or mother exposure to morphine also causes a significant decrease in hippocampus weight and concentration level of glutamate in the hippocampus. The mechanism(s) of morphine toxicity on the evolutionary phase of the gestational period and offspring learning and memory is largely unknown and needs further investigation, but perhaps the adverse effects of father morphine dependency before mating on children's learning may be transferred through the semen (19). In this regard, many effective drugs that include morphine are not mutagenic but these chemical agents often cause a minor but significant change in physiological parameters, behaviors and neuroendocrine parameters (20). However, more studies are needed to explain these observations. As far as we know, there is no data showing the impact of parent dependency on offspring’s spatial learning & memory and the results of this study are among the first reports showing the adverse effects of father and parent dependency on children’s learning & memory. Since the underlying mechanisms are not yet defined, clarification by further investigations is required. 

Prenatal opioid exposure consistently impairs learning and memory in a group of spatial tasks (15, 21), and causes opioid-induced defects in the development of the central nervous system and its structural and neurobehavioral consequences (5, 22). ,Previous studies have reported that opioids cross rapidly through the placenta and may increase the incidences of fetal adverse effects (23, 24). Maternal morphine consumption has been shown to result in physical and neurobehavioral defects, neurobiological changes (24), Pre- and postsynaptic alterations in the septohippocampal cholinergic innervations (25) in fetus and offspring. Mice and rats prenatally exposed to heroin revealed alterations in basal and carbachol-stimulated hippocampal Protein kinase C (PkC) activity and impairments in hippocampal long-term potentiation (LTP); which play an important role in hippocampal related behavioral abilities (5, 25). 

Previous reports revealed that prenatal opioid administration to pregnant rats transiently down-regulates mu opioid receptors in the neonatal and maternal brain (26). Hammer *et al* (1991) suggest that morphine exposure has selective regional effects on mu-receptor ontogeny in rat brains (27). Hammer *et al* (1991) reported that morphine reduces mu-receptor binding density in the pre-optic area of the hypothalamus, but not the primary somatosensory cortex. Mu-Receptor density was greater in the medial pre-optic area of females than males, and in superficial layers of cortex in males than females (27). 

Based on screening tests and interview data, prenatal exposure to opioids in pregnant women might be responsible for lower birth weight, high neonatal mortality, impaired psychomotor development and behavioral abnormalities (8, 23, 28). On the other hand, we observed a significant increase in the mean swimming speed in the MWM during the training and probe trials in both opium-exposed parents a fathers male offspring. An increase in the mean swimming speed may due to behavioral abnormalities, lack of attention, hyperactivity, aggressiveness and lack of social inhibition (8, 23, 28). However, we don’t know its exact mechanism(s) but it is in accordance with previous evidence showing that morphine induces the hyperactivation of the mesolimbic dopaminergic system, which results in increases in locomotor activity (29). 

## Conclusion

In summary, the results of this study showed that adult male rats that were born from opium dependent father, mother / both parents showed deficits in spatial learning or memory in Morris water maze test. The precise mechanism(s) by which prenatal opioid exposure impairs postnatal learning & memory remain largely unknown and further research is needed to elucidate the underlying mechanism(s).
